# Graves’ disease-related reversible intracranial arteriopathy presenting with ischemic stroke: A case report and literature review

**DOI:** 10.1097/MD.0000000000050067

**Published:** 2026-07-31

**Authors:** Wenjiang Wu, Jiangyun Xiong, Shimei Yin, Zhiyin Cai, Weili Wang, Xiaojing Guo

**Affiliations:** a Department of Neurology, Huishui County People’s Hospital, Huishui, Guizhou, China; b Department of Neurology, The Affiliated Suzhou Hospital of Nanjing Medical University, Suzhou, Jiangsu, China.

**Keywords:** case report, Graves’ disease, intracranial arteriopathy, ischemic stroke, thyrotoxicosis

## Abstract

**Rationale:**

Graves’ disease (GD) may be associated with ischemic stroke through nonatherosclerotic intracranial arteriopathy during thyrotoxicosis. However, the optimal antithrombotic regimen and treatment duration for this condition remain uncertain.

**Patient concerns:**

A 38-year-old man presented approximately 6.5 hours after the last known well with sudden dysarthria, aphasia, and right-sided weakness. His National Institutes of Health Stroke Scale (NIHSS) score was 9 on admission.

**Diagnoses:**

Brain magnetic resonance imaging demonstrated acute ischemic infarcts, and magnetic resonance angiography revealed left middle cerebral artery M2 stenosis. In the setting of overt Graves’ thyrotoxicosis and no identified alternative stroke source, the findings supported a diagnosis of Graves’ disease-related reversible intracranial arteriopathy presenting with ischemic stroke.

**Interventions:**

The patient received dual antiplatelet therapy with aspirin 100 mg and clopidogrel 75 mg daily for 21 days, together with atorvastatin and antithyroid treatment with thiamazole. The thiamazole dosage was subsequently adjusted according to serial thyroid function tests.

**Outcomes:**

The patient’s neurological deficits improved rapidly, and his NIHSS score decreased to 1 at discharge. Biochemical euthyroidism was achieved approximately 5 months after treatment initiation. At 12 months, magnetic resonance angiography demonstrated marked reversal of the left M2 stenosis. At the latest follow-up at 24 months, the patient was neurologically intact and remained free of recurrent ischemic or hemorrhagic events.

**Lessons:**

Graves’ disease-related reversible intracranial arteriopathy should be considered in young patients presenting with ischemic stroke, intracranial arterial stenosis, and thyrotoxicosis. This case suggests that short-term dual antiplatelet therapy may be considered as early bridging treatment in selected patients, whereas long-term management should prioritize sustained thyroid control and clinical and vascular imaging follow-up. Further evidence is required to establish the optimal antithrombotic regimen, treatment duration, and criteria for discontinuation.

## 1. Introduction

Graves’ disease (GD) has been linked to ischemic stroke not only via cardioembolism, for example atrial fibrillation, but also through non-atherosclerotic intracranial arteriopathy during thyrotoxicosis. This arteriopathy may manifest as reversible vasoconstriction or stenosis, and in a subset of progressive moyamoya-like narrowing, commonly involving the terminal internal carotid artery and extending to the middle cerebral artery (MCA).^[1,2]^

The proposed mechanisms include heightened sympathetic activity, endothelial dysfunction, oxidative stress, and immune-mediated injury in the setting of excess thyroid hormone.^[3,4]^ Observational data also suggest a correlation between thyroid autoantibodies and intracranial large-artery stenosis in young stroke patients, implying an immunologic contribution to vessel injury and recovery.^[5]^

Clinically, GD-related reversible intracranial stenosis may overlap with reversible cerebral vasoconstriction syndrome (RCVS), presenting with multifocal, segmental narrowing and ischemic or hemorrhagic complications. Differential diagnoses include atherosclerosis, primary central nervous system angiitis, arterial dissection, and moyamoya syndrome.^[6]^ Management centers on restoring euthyroidism with individualized vascular care; currently, no consensus or guideline defines a uniform antithrombotic strategy or duration for this phenotype.^[7]^ In RCVS-oriented practice, trigger removal and symptomatic vasodilator therapy, such as calcium-channel blockers, are typically prioritized, with decisions guided by serial vascular imaging.^[8,9]^ Here, we report a case and provide a focused literature review to highlight practical points regarding antithrombotic duration and explicit criteria for discontinuation in this setting.

## 2. Case presentation

### 2.1. Hospital day 1: admission

A 38-year-old right-handed man without hypertension or diabetes developed sudden dysarthria, slurred speech with impaired repetition, psychomotor slowing, and right-sided weakness, with reduced right-hand grip and an inability to stand or walk with the right leg, on April 5, 2023. The interval from the last known well to hospital arrival was approximately 6.5 hours. On admission, the NIHSS score was 9, with subscores of 2 for level-of-consciousness questions, 1 for facial palsy, 2 for the right arm, 2 for the right leg, and 2 for aphasia. Neurological examination revealed right lower facial weakness, characterized by flattening of the right nasolabial fold and deviation of the mouth to the left. Tongue protrusion was midline, and the remaining cranial nerve functions were normal. Muscle tone was normal throughout. Strength was 3/5 in the right upper and lower extremities and 5/5 in the left extremities on the Medical Research Council (MRC) scale. The right Babinski sign (extensor plantar response) was observed. Random capillary glucose was 10.9 mmol/L; complete blood count, electrolytes, renal and hepatic panels, and coagulation studies were unremarkable. Non-contrast head CT (NCCT) revealed a left parieto-occipital encephalomalacia consistent with a chronic infarct. In accordance with contemporaneous institutional practice, he was not treated with intravenous thrombolysis and did not undergo endovascular evaluation because the time from last known well to hospital arrival exceeded 6 hours. The attending physician favored a large-artery atherosclerotic mechanism and immediately initiated dual antiplatelet therapy (DAPT) with oral aspirin 100 mg and clopidogrel 75 mg, as well as atorvastatin 20 mg daily.

### 2.2. Hospital day 2: key neuroimaging

**Figure 1.**
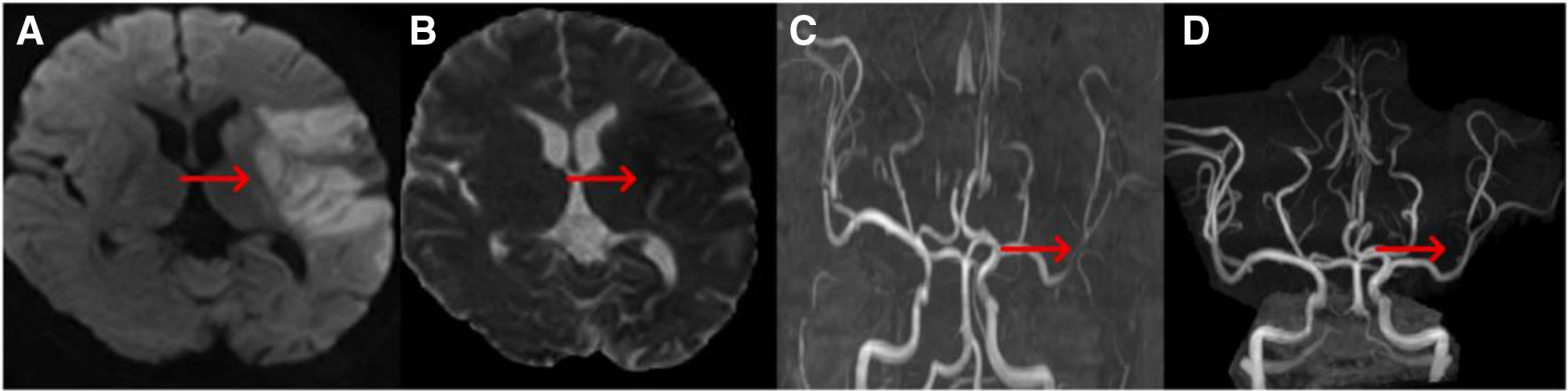
Admission and 12-month follow-up neuroimaging. (A) Axial DWI demonstrates hyperintense infarcts in the left temporal, basal ganglia, and parietal regions, with (B) corresponding hypointensity on the ADC map. (C) Baseline MRA reveals moderate-to-severe stenosis of the left MCA M2 segment with attenuation of distal branches. (D) Follow-up MRA at 12 months shows luminal recovery at the prior M2 stenotic segment. ADC = apparent diffusion coefficient, DWI = diffusion-weighted imaging, MCA = middle cerebral artery, MRA = magnetic resonance angiography.

On hospital day 2, diffusion-weighted magnetic resonance imaging (DWI) showed scattered acute infarcts in the left temporal lobe, basal ganglia, and parietal lobe, with corresponding low signal on apparent diffusion coefficient (ADC) maps (Fig. 1A, B). Magnetic resonance angiography (MRA) on the same day demonstrated moderate-to-severe stenosis of the M2 segment of the left MCA, with reduced visualization of distal branches (Fig. 1C). On that day, his neurologic deficits improved modestly, and the NIHSS score decreased to 7, composed of 2 for level-of-consciousness questions, 1 for facial palsy, 1 for the right arm, 1 for the right leg, and 2 for aphasia.

### 2.3. Hospital days 1 to 3: etiologic evaluation and treatment

Holter monitoring showed sinus rhythm with occasional premature atrial beats and a brief run of supraventricular tachycardia, but no sinus tachycardia, atrial fibrillation, or atrial flutter was detected. A 24-hour ambulatory blood pressure monitoring did not reveal hypertension. Carotid duplex ultrasonography, transthoracic echocardiography, transthoracic echocardiography with agitated-saline contrast (bubble study), and lower-limb venous ultrasonography revealed no significant abnormalities. Thyroid function tests revealed free triiodothyronine (FT3) of 30.97 pmol/L, free thyroxine (FT4) >100 pmol/L, and a markedly suppressed thyroid-stimulating hormone (TSH) level below 0.005 mIU/L, consistent with overt thyrotoxicosis. Thyroid autoantibodies were markedly elevated: thyroid peroxidase antibody (TPOAb) >600 IU/mL, TSH receptor antibody (TRAb) 6.82 IU/L, and thyroglobulin antibody (TGAb) 16.20 IU/mL. Thyroid ultrasonography demonstrated diffuse enlargement with coarse echotexture and marked hypervascularity.

Considering the unilateral M2 stenosis with multifocal acute infarcts, the presence of biochemical thyrotoxicosis with autoimmunity, and the absence of cardioembolic or atherosclerotic sources, we favored a GD-related non-atherosclerotic intracranial arteriopathy with reversible stenosis as the underlying stroke mechanism. Accordingly, we continued DAPT with aspirin 100 mg plus clopidogrel 75 mg for a 21-day course, together with atorvastatin 20 mg daily, and initiated antithyroid therapy with thiamazole under endocrinology supervision. Supportive care, such as hydration and hemodynamic optimization, was provided as clinically indicated.

### 2.4. Hospital day 14: discharge

The neurologic deficits continued to improve. At discharge, the NIHSS score was 1, entirely attributable to facial palsy (1 point), and the modified Rankin Scale (mRS) score was 1. On examination, muscle strength was MRC grade 5−/5 in the right upper and lower limbs and grade 5/5 in the left upper and lower limbs. The right Babinski sign remained present.

### 2.5. Post-discharge follow-up

All antithrombotics were discontinued on day 21, at the completion of the DAPT window, whereas antithyroid therapy with thiamazole was continued and titrated under endocrinology supervision. Serial thyroid function tests evolved from frank thyrotoxicosis through a TSH-suppressed phase, reaching biochemical euthyroidism by approximately 5 months. A transient hypothyroid pattern consistent with overtreatment occurred and resolved after dose reduction, with sustained euthyroidism thereafter. Thiamazole dosing was guided by thyroid function tests; the dose was briefly escalated to 30 mg once daily and then tapered stepwise to 20 mg, 10 mg, and 5 mg once daily.

**Figure 2.**
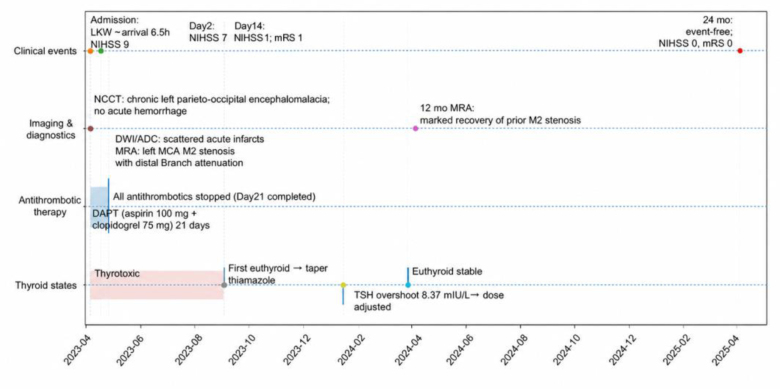
Timeline of the patient’s events. ADC = apparent diffusion coefficient, ATD = antithyroid drug, DWI = diffusion-weighted imaging, LKW = last known well, MCA = middle cerebral artery, MRA = magnetic resonance angiography, mRS = modified Rankin Scale, NIHSS = National Institutes of Health Stroke Scale, TSH = thyroid-stimulating hormone.

**Figure 3.**
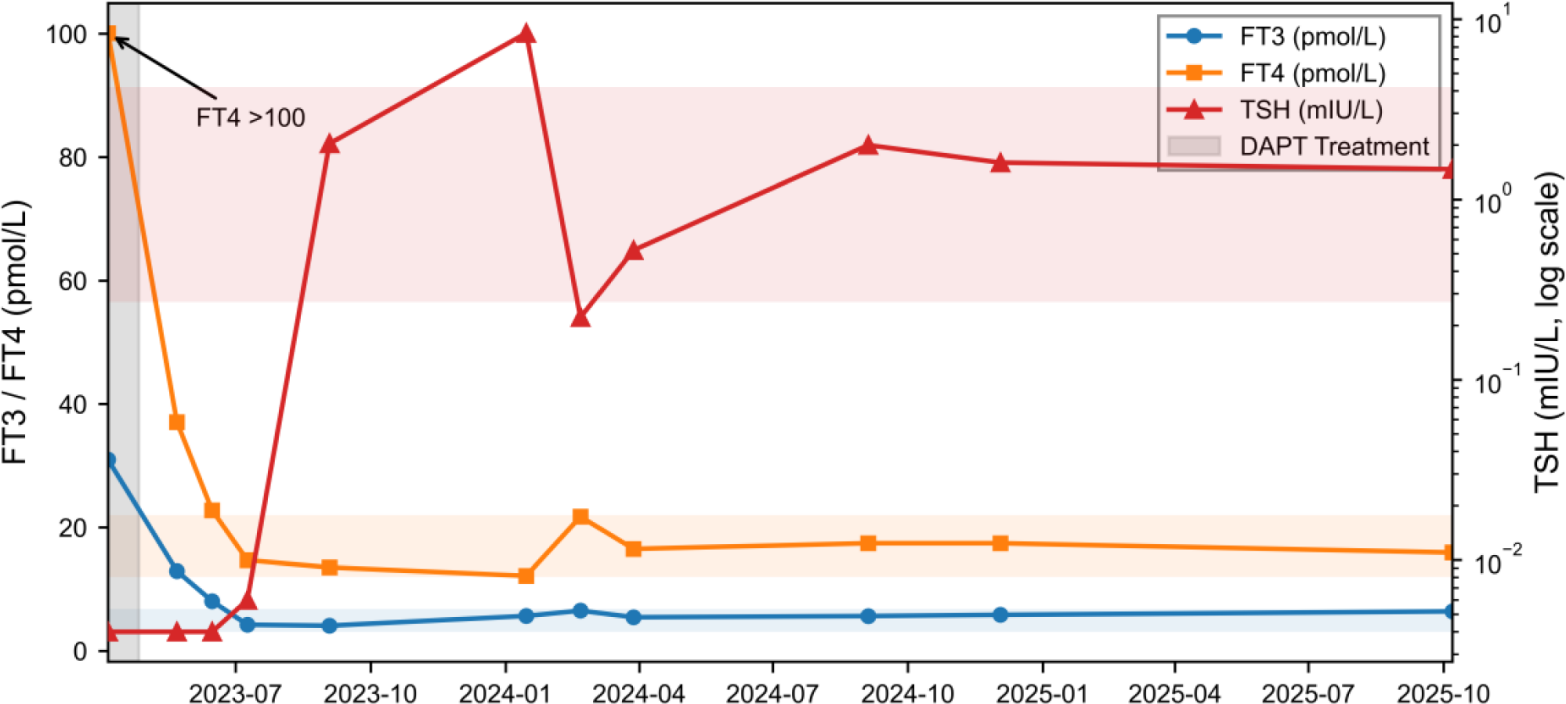
Longitudinal trends in thyroid function during and after DAPT treatment. The left *y*-axis represents FT3 (blue circles) and FT4 (orange squares) levels in pmol/L. The right *y*-axis represents TSH levels (red triangles) in mIU/L on a logarithmic scale. The shaded horizontal bands, corresponding to the plot colors, indicate institutional reference ranges: FT3 (3.10–6.80 pmol/L), FT4 (12.00–22.00 pmol/L), and TSH (0.27–4.20 mIU/L). The vertical gray shaded region marks the duration of dual antiplatelet therapy (DAPT; April 5 to April 26, 2023). An annotation indicates the initial FT4 level exceeding the measurement limit (>100 pmol/L). DAPT = dual antiplatelet therapy, FT3 = free triiodothyronine, FT4 = free thyroxine, TSH = thyroid-stimulating hormone.

At 12 months, MRA showed marked reversal of the left M2 stenosis. At the latest follow-up (24 months), the patient was neurologically intact, with NIHSS score 0 and mRS score 0, and had no recurrent ischemic or hemorrhagic events. Figures 2 and 3 summarize the clinical timeline, DAPT and subsequent antithrombotic discontinuation, thiamazole dose adjustments, and thyroid function test trajectories. The patient expressed relief that the cause of the stroke was identified and treated without the need for lifelong antithrombotic medication. He reported a full return to his daily activities and was satisfied with the clinical outcome.

## 3. Discussion

Graves’ disease (GD) and thyrotoxicosis can precipitate ischemic stroke through non-atherosclerotic, inflammatory or vasoreactive intracranial narrowing.^[2,10,11]^ Guidance on diagnostic work-up, antithrombotic duration and stopping rules remains limited.^[1,7,12,13]^ Here, we report a single-timeline case illustrating the sequence of short-course DAPT, continued endocrine treatment, and subsequent angiographic reversal. The patient received DAPT for 21 days as early bridging therapy. Antithrombotics were discontinued after this planned early high-risk window, whereas antithyroid therapy was continued until biochemical euthyroidism was achieved. At the 24-month follow-up, the patient remained event-free, with marked luminal recovery on vascular imaging.

### 3.1. Clinical phenotype and differentials

A 38-year-old man, during biochemical thyrotoxicosis, had brain magnetic resonance imaging (MRI) showing multiple scattered hyperintense lesions in the left temporal lobe, basal ganglia, and parietal lobe. MRA demonstrated moderate-to-severe stenosis of the left MCA M2 segment with reduced distal branches. The imaging pattern did not meet criteria for moyamoya syndrome (MMS), as there was no bilateral involvement of the terminal internal carotid arteries (ICAs) or classic basal collaterals. The clinical and imaging features also argued against reversible cerebral vasoconstriction syndrome (RCVS). There was no thunderclap headache or beaded multisegmental narrowing, making RCVS less likely.^[6,8]^ Primary angiitis of the central nervous system (PACNS) was not supported by the clinical profile, which was characterized by a non-insidious course and an absence of systemic immune activation.^[14]^ The burden of vascular risk factors was low, and there were no signs of arterial dissection or typical intracranial atherosclerosis. The constellation of active thyrotoxicosis plus unilateral moderate-to-severe M2 stenosis with distal branch paucity and multiple scattered ischemic-appearing lesions is compatible with GD-related reversible intracranial arteriopathy (GD-RICAS).^[6,14,15]^ Previous reports have described angiographic improvement after restoration of euthyroidism,^[1,16]^ and vessel wall imaging (VWI) may show inflammatory enhancement in selected cases.^[17,18]^ Taken together, these findings align with the young-onset spectrum in which non-atherosclerotic intracranial arteriopathies and endocrine–immune drivers are proportionally more common.^[19,20]^

### 3.2. De-escalation approach and risk-benefit

We adopted a short course of intensive antithrombotic therapy with early de-escalation because the etiology pointed to GD-RICAS rather than intracranial atherosclerotic disease, RCVS, or MMS. The patient was young, with a low vascular risk-factor burden and, during autoimmune thyrotoxicosis, had unilateral moderate-to-severe stenosis of the M2 segment of the left MCA with distal branch paucity and multiple scattered ischemic lesions in the left temporal lobe, basal ganglia, and parietal lobe. These features are more consistent with a non-atherosclerotic inflammatory or vasoreactive mechanism than with a platelet-driven atherothrombotic mechanism.^[6,8]^ In this context, antithrombotic agents do not modify the underlying disease and, with prolonged exposure, mainly accrue bleeding risk. In contrast, randomized data show that short-term DAPT provides most of its benefit within the first 21 days. Extending treatment beyond this period mainly increases bleeding risk.^[20–25]^ We, therefore, used DAPT only as a 3-week bridge to cover the early high-risk period. Endocrine correction is a disease-modifying therapy for GD-RICAS, and immunomodulation is added when vessel-wall inflammation is present. The biological plausibility of this mechanism is strong, as thyroid hormones alter endothelial function and vascular smooth muscle reactivity, reduce systemic vascular resistance, and amplify sympathetic responses, thereby disturbing hemodynamics and vascular tone.^[26]^ VWI may show inflammatory enhancement in selected cases.^[27]^ Previous reports have noted that angiographic recovery typically lags behind biochemical control. It usually improves gradually over several months and tends to mirror changes in thyroid function. These observations support a planned de-escalation strategy in which short-term antithrombotic therapy is used as early bridging treatment, while longer-term management depends on endocrine control, clinical stability, and follow-up vascular imaging.^[4]^

### 3.3. Thyroid–vascular coupling and transient TSH overshoot

Figure 2 illustrates the coupling between the thyroid indices and key therapeutic and imaging milestones. During follow-up, a single transient TSH overshoot to 8.37 mIU/L occurred while FT3/FT4 were in the low-normal range, consistent with hypothalamic–pituitary–thyroid (HPT) axis lag and mild over-suppression. After a minor dose adjustment, thyroid function stabilized within several weeks and remained normal during long-term follow-up. This brief fluctuation did not alter our management rule; DAPT, used only as a bridge, had already been discontinued at approximately 3 weeks. Importantly, biochemical control typically precedes angiographic reversal, which often unfolds over months.^[1,16]^ Accordingly, antithrombotic decisions should not hinge on a single TSH value but should integrate FT3/FT4 trends, imaging, and clinical symptoms/signs.^[4,28]^

### 3.4. Relation to prior literature

To make the evidence and strategy auditable and comparable, we compiled all retrieved studies on GD-RICAS in Table 1 with harmonized, decision-focused fields. Across case series and reports, antiplatelet therapy was commonly used, but the specific agents, duration, and criteria for discontinuation were seldom reported, limiting the ability to synthesize findings across studies.^[7,13]^ In contrast, sustained endocrine control consistently aligns with better outcomes, whereas discontinuation or nonadherence to antithyroid therapy tracks recurrence or progression, including fatal courses when thyrotoxicosis remains uncontrolled.^[7,13,29-31]^ Imaging findings further support a thyroid-driven, often reversible arteriopathy. Angiographic improvement usually appears around 6 to 12 weeks and tends to lag behind biochemical euthyroidism. Restenosis may occur with thyroid relapse, supporting the use of antiplatelet therapy as early bridging rather than as disease-modifying treatment.^[16,32]^ VWI reveals phenotypic heterogeneity. In addition to reversible vasoreactive patterns, a subset shows inflammatory or vasculitic wall enhancement, with luminal improvement associated with corticosteroids with or without immunosuppressants. Aspirin is generally used as an adjunct.^[1,33]^ Early reports suggest that, in large-artery occlusion attributed to thyroid disease, revascularization should be considered only after euthyroid correction and then only if symptoms or perfusion continue to worsen. Outcomes appear similar with antithyroid therapy alone and with antithyroid therapy combined with revascularization, supporting individualized rather than routine use of surgery in this stroke subtype.^[7,34]^

**Table 1:** Prior literature on Graves’ disease-related reversible intracranial arteriopathy (GD-RICAS): decision-focused summary.

First author	Year	Design (n)	Age	Gender	Key cerebrovascular imaging	Thyroid status at index event (antibodies)	Antithrombotic therapy (with duration)	Endocrine or immune therapy; achieved normal thyroid function (yes/no)	Angiographic reversibility (yes/no; wk)	Outcome/recurrence	Country
Nakamura	2003	Two-case series (n = 2)	23, 54	Female/female	Multiple intracranial arterial stenoses around the circle of Willis; no moyamoya-type basal collaterals; MRI: infarcts (case 1: right temporoparietal; case 2: left frontal)	Case 1: Thyrotoxicosis (TRAb 30%); Case 2: subclinical hyperthyroidism (near-euthyroid; TSH 0.1 mIU/L). Thyroid antibodies: as stated; others not reported	Not reported	Antithyroid drugs (thiamazole) → bilateral STA–MCA bypass with EMS after achieving euthyroid; yes	No; ~24 wk; native luminal stenosis not reversed; bypass collaterals developed	Symptoms resolved; returned to normal daily life; no recurrence reported	Japan
Utku	2004	Case report (n = 1)	45	Female	MRA: severe bilateral distal ICA and distal basilar artery stenoses; moyamoya-type basal collaterals	Thyrotoxicosis (FT3 13.8 pg/dL; FT4 5.07 ng/dL; TSH 0.001 mIU/L). Thyroid antibodies: TgAb 418 IU/mL and TPOAb 1000 IU/mL, positive	No	Intravenous methylprednisolone (pulse) + plasmapheresis; euthyroid status not reported	Yes; ~12 wk; MRA normalized	Neurological status markedly improved; no recurrence reported	Turkey
Yamashita	2005	Case report (n = 1)	29	Female	Initial MRA: bilateral distal ICA and proximal ACA/MCA stenoses; DSA: right distal ICA with mild right proximal ACA and left proximal MCA stenoses; no moyamoya-type basal collaterals	Thyrotoxicosis (TSH 0.03 mIU/L; FT4 6.0 ng/dL; FT3 25.19 ng/dL). Thyroid antibodies: TgAb 480% (elevated)	Aspirin 81 mg/d; duration not reported	Thiamazole 15 mg/d; yes (remission; FT4/FT3 normalized)	Yes; timing not reported; terminal ICA stenosis improved on follow-up MRA	Symptoms resolved; no recurrence reported	Japan
Ohba	2011	Case report + literature review (index n = 1)	34	Female	Severe bilateral terminal ICA stenosis with moyamoya-type basal collaterals; MRI: lacunar infarcts; SPECT: bilateral hypoperfusion	Hypothyroidism (FT3 1.1 pg/mL; FT4 0.4 ng/dL; TSH 53.8 mIU/L). Thyroid antibodies: not reported	Antiplatelet therapy; agent not specified; duration not reported	Subtotal thyroidectomy → thiamazole + levothyroxine; yes (euthyroid maintained)	No; ~12 wk; native terminal ICA stenoses persisted; bypass inflow present	Symptoms improved; occasional TIAs on 2-yr follow-up; euthyroid maintained	Japan
Cheon	2014	Two-case series (n = 2)	12, 15	Female/female	Case 1: MRA: right MCA stenosis; Case 2: terminal ICA and proximal MCA stenoses with moyamoya-type basal collaterals (typical moyamoya pattern)	Thyrotoxicosis. Case 1: T3 213.36 ng/dL, FT4 1.64 ng/dL, TSH 0.24 mIU/L, TRAb 112.66 U/L (as reported). Case 2: FT3 1.07 ng/mL, FT4 40.99 ng/dL, TSH 0.01 mIU/L, TRAb 4.1 U/mL (as reported). Thyroid antibodies: as detailed above	No	Thiamazole (case 1: 20 mg/d; case 2: dose not stated); yes (euthyroid achieved)	Not reported; EDAS planned (elective)	Improved; no further syncope (case 1); right-sided strength recovered (case 2); no recurrence reported	South Korea
Ni	2014	Series (n = 12)	Mean 33.3 ± 12.7 (range 11–55)	Female 12/12	Terminal (distal) ICA stenosis/occlusion with or without proximal ACA/MCA involvement; often bilateral; moyamoya-type basal collaterals common; occasional PCA stenosis; MRI (DWI): acute/subacute infarcts mainly in frontal/watershed regions; HR-VWI normal in 2 cases	Thyrotoxicosis in 11/12 at stroke/TIA; Thyroid antibodies: positive in 12/12 (TPOAb and/or TgAb)	Antiplatelet therapy with or without anticoagulation; duration not reported	Antithyroid drugs; yes (euthyroid achieved)	Not reported; limited follow-up	Most improved with sustained antithyroid therapy; 2 relapses after stopping antithyroid drugs	China
Ku	2015	Case report (letter) (n = 1)	42	Female	Progressive moyamoya vasculopathy; MRI: initial small ischemic lesions in bilateral basal ganglia and periventricular white matter; at 1 yr, new small acute infarct in the left caudate with progression; on final admission, multiple large hemispheric infarcts with progressive transtentorial herniation	Uncontrolled thyrotoxicosis (FT3 5.5; FT4 14.0; TSH < 0.03 mIU/L; units as reported). Thyroid antibodies: not reported	No	Therapy not reported; No (euthyroid not achieved)	No; progressive over ~52 wk	Death (fatal ischemic stroke with herniation)	South Korea
Yin	2015	Case report (n = 1)	17	Male	Multiple infarcts in the left MCA territory; MRA: severe unilateral M1 stenosis; HR-VWI: non-atherosclerotic features	Thyrotoxicosis (FT3 13.706 pmol/L; FT4 42.25 pmol/L; TSH 0.003 mIU/L). Thyroid antibodies: TRAb 5.66 IU/L and TPOAb 54.44 IU/mL, positive	Antiplatelet therapy; agent not specified; duration not reported	Antithyroid drugs (standard therapy); yes (labs normalized at 1 yr)	Not reported	Improved; NIHSS 0 at 1 yr; no recurrence reported	China
Choi	2016	Case report (n = 1)	31	Female	CTA: complete occlusion of the left cavernous/supraclinoid ICA; severe right ICA stenosis near the bifurcation with M1/A1 stenoses; PCom collateral supply to the anterior circulation; MRI (DWI/FLAIR): acute left frontal cortical infarct; follow-up MRA/MRV: bilateral cavernous ICA occlusions, right MCA occluded, left MCA diminutive	Severe thyrotoxicosis (TSH < 0.005 mIU/L; total T4 23.07 μg/dL; total T3 > 651 ng/dL). Thyroid antibodies: not reported	Antiplatelet therapy; agent not specified; duration not reported	Propranolol 40 mg PO q8h + thiamazole 10 mg PO q8h (initiated late); no (euthyroid not achieved)	No; progressive occlusions	Death (decompressive craniotomy; complications/infection while thyroid dysfunction persisted)	United States
Shah	2016	Case series + literature review (n = 8)	Mean 32 (range 19–48)	Female 8/8	Bilateral terminal ICA stenosis with or without proximal MCA involvement; ACA involvement common; ischemic lesion extent assessed by MRA and DWI	Thyrotoxicosis in 8/8 at acute infarct (TSH undetectable; total T4 elevated, mean 9.9 ± 7.3 ng/dL). Thyroid antibodies: positive in 5/8 (TRAb, TPOAb, TgAb)	Antiplatelet therapy; agent not specified; duration not reported	Antithyroid drugs (thiamazole or propylthiouracil) with or without oral corticosteroids and propranolol; euthyroid achieved in reported cases	Not reported; not systematically assessed	Most improved with medical therapy; progression or contralateral stroke with poor adherence/uncontrolled thyrotoxicosis; some required EC–IC bypass; at 1-yr follow-up, no additional strokes reported	United States
Ito	2019	Case report + literature review (n = 1)	37	Female	Left MCA-territory cortical–subcortical infarcts (DWI); initial MRA near-normal left ICA, then progressive long-segment left ICA (proximal→distal) with or without MCA stenosis; contrast-enhanced HR-VWI (3D T1-weighted): smooth concentric circumferential wall thickening with diffuse enhancement; DSA confirmed progressive ICA stenosis and later collateral development	Thyrotoxicosis (FT3 10.58 pg/mL; FT4 2.70 ng/dL; TSH 0.01 mIU/L). Thyroid antibodies: TPOAb 148 IU/mL and TRAb 8.3 IU/mL, positive	Heparin → warfarin 4 mg/d; relapse: argatroban; later dual antiplatelet therapy (clopidogrel 75 mg/d + aspirin 100 mg/d); duration not reported	Potassium iodide 150 mg/d (initially, leukopenia) → thiamazole 15 mg/d; intravenous methylprednisolone (pulse) → prednisolone 1 mg/kg/d + methotrexate (titrated to 14 mg/wk); yes (euthyroid achieved)	Yes (partial); ~78–104 wk; vessel-wall enhancement persisted on CE HR-VWI (3D T1-weighted)	Attacks ceased after intravenous methylprednisolone (IVMP) plus antiplatelets; cerebral flow improved on MRA/DSA; slight lumen improvement at terminal ICA; no surgery	Japan
Hidaka	2020	Case report (n = 1)	31	Female	MRA: bilateral terminal ICA and proximal MCA stenoses; MRI: acute infarcts in the right frontal and parietal lobes; follow-up: improvement, then mild restenosis with thyroid worsening	Thyrotoxicosis (FT3 > 12.30 pg/mL; FT4 3.04 ng/dL; TSH 0.00 mIU/L). Thyroid antibodies: not reported	Aspirin + cilostazol; duration not reported	Potassium iodide 20 mg/d + thiamazole 15 mg/d; yes (euthyroid by ~2 mo); thyroid function worsened at 6 mo	Yes; ~8–16 wk; mild bilateral MCA restenosis at ~26 wk with thyrotoxic flare	Clinical symptoms resolved; no recurrence reported; mild restenosis on follow-up	Japan
Sasaki	2023	Case report (n = 1)	35	Female	Bilateral MCA stenosis with prominent leptomeningeal collaterals (“ivy sign”); perfusion deficit	Thyrotoxicosis (FT4 > 4.79 ng/dL; FT3 > 15.20 pg/mL); Thyroglobulin > 127.82 ng/mL. Thyroid antibodies: TPOAb > 998.5 IU/mL and TRAb > 7.53 IU/L, positive	No	Potassium iodide 20 mg/d + thiamazole 15 mg/d; yes (fT3/fT4 normalized; symptom improvement)	No; ~50 wk (352 d); MRA lumen unchanged; FLAIR “ivy sign” and perfusion improved	Cognitive symptoms markedly improved; no recurrence reported	Japan
Pierman	2024	Case report (n = 1)	25	Female	MRI: infarcts in the right MCA territory and left deep MCA (centrum semiovale)	Thyrotoxicosis (TSH < 0.01 mIU/L; FT4 59.61 pg/mL; FT3 25.17 pg/mL). Thyroid antibodies: TRAb 10.4 IU/L and TPOAb 485 IU/mL, positive	LMWH (enoxaparin 4000 IU) → aspirin 80 mg daily; duration not reported	Thiamazole 60 mg/d + propranolol 20 mg/d; yes (thyroid hormones normalized by 1 mo); thiamazole continued > 18 mo then stopped	Stable; lumen reversal not reported	Deficits resolved by 1 mo; TIA at 3 mo with no new lesions; 10-yr follow-up: no recurrence of hyperthyroidism or stroke; cognitive symptoms improved with rehabilitation	Belgium

ACA = anterior cerebral artery, CE = contrast-enhanced, CTA = computed tomographic angiography, DSA = digital subtraction angiography, DWI = diffusion-weighted imaging, EC–IC = extracranial–intracranial, EDAS = encephaloduroarteriosynangiosis, EMS = encephalomyosynangiosis, FLAIR = fluid-attenuated inversion recovery, GD-RICAS = Graves’ disease-related reversible intracranial arteriopathy, HR-VWI = high-resolution vessel-wall imaging, ICA = internal carotid artery, LMWH = low-molecular-weight heparin, MCA = middle cerebral artery, MRA = magnetic resonance angiography, MRV = magnetic resonance venography, NIHSS = National Institutes of Health Stroke Scale, PCA = posterior cerebral artery, PCom = posterior communicating artery, SPECT = single-photon emission computed tomography, STA–MCA = superficial temporal artery–middle cerebral artery, TIA = transient ischemic attack, TPOAb = antithyroid peroxidase antibody, TRAb = TSH receptor antibody, TgAb = antithyroglobulin antibody.

Conventions: Units are shown as reported in the source articles; “~” denotes approximate values; wk = weeks, mo = months, yr = years; PO q8h = orally every 8 hours.

In this context, we used a 21-day course of DAPT to bridge the first 2 to 3 high-risk weeks and discontinued antithrombotic therapy after the completion of this planned early treatment window. Long-term secondary prevention relied on endocrine control and regular follow-up vascular imaging. Table 1 summarizes and compares the key decision elements and case-level details.

### 3.5. Strengths and limitations

This report provides detailed longitudinal clinical, biochemical, and vascular imaging follow-up over 24 months, allowing the temporal relationship between thyroid control and arterial recovery to be assessed. Nevertheless, several limitations should be acknowledged. First, this was a single-patient observation, and a causal relationship between Graves’ disease and the intracranial arteriopathy cannot be definitively established. Second, digital subtraction angiography and high-resolution vessel wall imaging were not performed. Therefore, the underlying vascular pathology could not be directly characterized. Third, the absence of an untreated comparator prevents the determination of the independent contributions of antiplatelet and antithyroid therapies to the favorable outcome. Further studies are needed to define the optimal antithrombotic regimen and treatment duration for this condition.

## 4. Conclusions

GD-RICAS likely reflects a combination of vascular reactivity and inflammation. Management should prioritize rapid correction of the endocrine abnormality, early but time-limited antithrombotic therapy with planned de-escalation, and regular clinical assessment with follow-up vascular imaging. Because the available literature is dominated by heterogeneous case reports and small series, future studies should use standardized data elements to define antithrombotic regimens and stopping rules.

## Acknowledgments

The authors thank the patient for agreement to the publication of the report.

## Author contributions

**Conceptualization:** Wenjiang Wu.

**Data curation:** Wenjiang Wu, Jiangyun Xiong, Shimei Yin, Zhiyin Cai, Weili Wang.

**Formal analysis:** Wenjiang Wu, Jiangyun Xiong.

**Investigation:** Shimei Yin, Zhiyin Cai, Weili Wang.

**Resources:** Shimei Yin, Weili Wang.

**Software:** Jiangyun Xiong, Shimei Yin.

**Supervision:** Wenjiang Wu, Xiaojing Guo.

**Validation:** Jiangyun Xiong, Shimei Yin, Zhiyin Cai, Weili Wang, Xiaojing Guo.

**Visualization:** Jiangyun Xiong.

**Writing – original draft:** Wenjiang Wu.

**Writing – review & editing:** Xiaojing Guo.
